# Supplementary material for: Priming Potato Plants with Melatonin Protects Stolon Formation under Delayed Salt Stress by Maintaining the Photochemical Function of Photosystem II, Ionic Homeostasis and Activating the Antioxidant System
Source: Int J Mol Sci. 2023 Mar 24;24(7):6134. doi: 10.3390/ijms24076134 (PMC10094597; doi:10.3390/ijms24076134)
Supplement: Supplementary file 1 [file ijms-24-06134-s001.zip › ijms-2273765-SI/Table S1.pdf]

Table S1. Primers used in the analysis and alignment of all studied genes.

Gene	Accession number	Primer sequence<br>F – forward primer<br>R – reverse primer	PCR product length
Reference genes			
EF1α	LOC102600998<br>LOC102600107	F – ATTGGAAATGGATATGCTCCA<br>R – TCCTTACCTGAACGCCTGTCA	101
Cyclophilin	LOC102586427	F – GCTCTTCGCCGATACCACTC<br>R – ACACGGTGGAAGGTTGAGC	120
Target genes			
P5CS1	LOC102582423	F – CTGCTCCTACATTTCTCTCCTTCG<br>R – CGCTCCTAATCTTCCAAGTGCT	160
PDH	LOC102588726<br>LOC102588395	F – ATAACTGCAATTTGTACACCA<br>R – GGCGAAAAGTGGAAGCG	117
PAL	LOC102596343<br>LOC102585357<br>LOC102585026<br>LOC102587888<br>LOC102596891	F – TCGCTGAAGTGATGAACGGAA<br>R – ATTGTGGAGATGTTCGGAGA	207
CHS1a	LOC102577871	F – CTTGTCCCCGATAGCGAA<br>R – AAATAGAGAGTTCCAGTCAGAT	162
APX1	LOC102586473<br>LOC102586145	F – CTGGTGTCGTTGCTGTTGAA<br>R – AAGATAAGAGGATTGGCGGTC	259
APX3	LOC102597356	F – CCTTCTTTAGGGATTACGCCAT<br>R – AGCCACAACAACACCTACAGCA	131
SOS1	LOC102602690	F – TTGCCTCACCATTCCATTG<br>R – TGATGCTTCTGTGTTGTGGAAG	138
NHX1	LOC102578176	F –TGTTGATCCCTTTCGACCAT<br>R – CCAAATAGGGGTCGCATAAA	111
NHX2	LOC102605641	F – TGTTACTGGCATTCTGGTTTAC<br>R – AATAGACAGAACAGTGACAGGA	124
NHX3	LOC102589621	F – GTCCATTACTGCCCTTGTTAT<br>R – AAATGATAGGTGGAAGAAGGTAA	131
