# Supplementary material for: Priming Potato Plants with Melatonin Protects Stolon Formation under Delayed Salt Stress by Maintaining the Photochemical Function of Photosystem II, Ionic Homeostasis and Activating the Antioxidant System
Source: Int J Mol Sci. 2023 Mar 24;24(7):6134. doi: 10.3390/ijms24076134 (PMC10094597; doi:10.3390/ijms24076134)
Supplement: Supplementary file 1 [file ijms-24-06134-s001.zip › ijms-2273765-SI/Table S2.pdf]

Table S2. Influence of melatonin on the photochemical activity of photosystem II of *Solanum tuberosum* leaves under saline conditions.

Treatment															
NaCl, mM	Melatonin, μM	Y (II)	%	ETR	%	qN	%	NPQ	%	Y (NO)	%	Y (NPQ)	%	F_v/F_m	%
0	0	0.60±0.02	100	47.56±1.76	100	0.33±0.04	100	0.40±0.06	100	0.29±0.01	100	0.12±0.02	100	0.81±0.01	100
125	0	0.37±0.05*	61	28.84±4.10*	61	0.46±0.09	137	0.52±0.14	129	0.42±0.02*	145	0.21±0.05*	186	0.47±0.05*	58
125	0.1	0.30±0.10*	51	23.73±8.34*	50	0.58±0.04*	175	0.78±0.14*	195	0.40±0.03*	137	0.30±0.07*	263	0.51±0.02*	63
125	1.0	0.41±0.04*	69	32.92±3.39*	69	0.55±0.07*	165	0.81±0.16*	200	0.33±0.03#	115	0.25±0.04*	221	0.62±0.04*,#	77
125	10.0	0.51±0.02*	86	40.32±1.67*,#	85	0.38±0.10	115	0.50±0.15	124	0.30±0.02#	103	0.20±0.01*	177	0.75±0.02*,#	92

*$p < 0.05$ compared to the control value; #$p < 0.05$ compared to «125 mM NaCl» value.
