# Supplementary material for: Priming Potato Plants with Melatonin Protects Stolon Formation under Delayed Salt Stress by Maintaining the Photochemical Function of Photosystem II, Ionic Homeostasis and Activating the Antioxidant System
Source: Int J Mol Sci. 2023 Mar 24;24(7):6134. doi: 10.3390/ijms24076134 (PMC10094597; doi:10.3390/ijms24076134)
Supplement: Supplementary file 1 [file ijms-24-06134-s001.zip › ijms-2273765-SI/Table S3.pdf]

Table S3. Influence of melatonin on the levels of *APX1* and *APX3* gene transcripts in *Solanum tuberosum* leaves under saline conditions.

Treatment					
NaCl, mM	Melatonin, μM	*APX1*	%	*APX3*	%
0	0	0.848±0.015	100	0.037±0.001	100
125	0	0.892±0.015	105	0.027±0.001*	75
125	0.1	0.486±0.026*^,#^	57	0.028±0.002*	77
125	1.0	0.586±0.041*^,#^	69	0.028±0.002*	78
125	10.0	0.651±0.032*^,#^	77	0.028±0.001*	77

*p < 0.05 compared to the control value; #p < 0.05 compared to «125 mM NaCl» value.
