# Supplementary material for: Priming Potato Plants with Melatonin Protects Stolon Formation under Delayed Salt Stress by Maintaining the Photochemical Function of Photosystem II, Ionic Homeostasis and Activating the Antioxidant System
Source: Int J Mol Sci. 2023 Mar 24;24(7):6134. doi: 10.3390/ijms24076134 (PMC10094597; doi:10.3390/ijms24076134)
Supplement: Supplementary file 1 [file ijms-24-06134-s001.zip › ijms-2273765-SI/Table S4.pdf]

Table S4. Influence of melatonin on the levels of *PAL* and CHS1α gene transcripts in *Solanum tuberosum* leaves under saline conditions.

Treatment					
NaCl, mM	Melatonin, μM	*PAL*	%	*CHS1α*	%
0	0	0.320 ± 0.011	100	0.0113 ± 0.0006	100
125	0	0.414 ± 0.009*	129	0.0039 ± 0.0003*	35
125	0.1	0.396 ± 0.004*	124	0.00~~1~~19 ± 0.0002#	17
125	1.0	0.132 ± 0.004*,#	41	0.0039 ± 0.0004*	34
125	10.0	0.232 ± 0.009*,#	72	0.0047 ± 0.0004*,#	42

*$p < 0.05$ compared to the control value; #$p < 0.05$ compared to «125 mM NaCl» value.
